# MEDREV (pharmacy-health psychology intervention in people living with dementia with behaviour that challenges): the feasibility of measuring clinical outcomes and costs of the intervention

**DOI:** 10.1186/s12913-020-5014-0

**Published:** 2020-03-02

**Authors:** Ian D. Maidment, Garry Barton, Niyah Campbell, Rachel Shaw, Nichola Seare, Chris Fox, Steve Iliffe, Emma Randle, Andrea Hilton, Graeme Brown, Nigel Barnes, Jane Wilcock, Sarah Gillespie, Sarah Damery

**Affiliations:** 1 School of Life and Health Sciences, Aston University, Birmingham, B4 7ET UK; 2 Norwich Clinical Trials Unit, University of East Anglia, Earlham Road, Norwich, Norfolk, NR4 7TJ UK; 3 Aston Health Research Innovation Cluster, Aston University, Birmingham, B4 7ET UK; 4 Norwich Medical School, University of East Anglia, Earlham Road, Norwich, Norfolk, NR4 7TJ UK; 5 Research Department of Primary Care & Population Health, University College London, Royal Free Campus, Rowland Hill St, London, NW3 2PF UK; 6 Birmingham and Solihull Mental Health NHS Foundation Trust, Research and Innovation Department, The Barberry, 25 Vincent Drive, Birmingham, B15 2FG UK; 7 Faculty of Health Sciences, University of Hull, Hull, HU6 7RX UK; 8 Birmingham and Solihull Mental Health NHS Foundation Trust, Unit 1, B1, 50 Summer Hill Road, Birmingham, B1 3RB UK; 9 Department of Clinical Healthcare, Faculty of Health and Life Sciences, Oxford Brookes University, Gipsy Lane Campus, Headington, Oxford, OX3 0FL UK; 10 Institute of Applied Health Research, College of Medical and Dental Sciences, University of Birmingham, Edgbaston, Birmingham, B15 2TT UK

**Keywords:** Dementia, Feasibility study, Behaviour that challenges, Psychotropics

## Abstract

**Background:**

People living with dementia in care homes frequently exhibit “behaviour that challenges”. Anti-psychotics are used to treat such behaviour, but are associated with significant morbidity. This study researched the feasibility of conducting a trial of a full clinical medication review for care home residents with behaviour that challenges, combined with staff training. This paper focusses on the feasibility of measuring clinical outcomes and intervention costs.

**Methods:**

People living with moderate to severe dementia, receiving psychotropics for behaviour that challenges, in care homes were recruited for a medication review by a specialist pharmacist. Care home and primary care staff received training on the management of challenging behaviour.

Data were collected at 8 weeks, and 3 and 6 months. Measures were Neuropsychiatric Inventory-Nursing Home version (NPI-NH), cognition (sMMSE), quality of life (EQ-5D-5 L/DEMQoL) and costs (Client Services Receipt Inventory).

Response rates, for clinical, quality of life and health economic measures, including the levels of resource-use associated with the medication review and other non-intervention costs were calculated.

**Results:**

Twenty-nine of 34 participants recruited received a medication review. It was feasible to measure the effects of the complex intervention on the management of behaviour that challenges with the NPI-NH. There was valid NPI-NH data at each time point (response rate = 100%). The sMMSE response rate was 18.2%. Levels of resource-use associated with the medication review were estimated for all 29 participants who received a medication review. Good response levels were achieved for other non-intervention costs (100% completion rate), and the EQ-5D-5 L and DEMQoL (≥88% at each of the time points where data was collected).

**Conclusions:**

It is feasible to measure the clinical and cost effectiveness of a complex intervention for behaviour that challenges using the NPI-NH and quality of life measures.

**Trial registration:**

ISRCTN58330068. Retrospectively registered, 15 October 2017.

## Background

The number of people living with dementia is rapidly increasing not just in the UK but internationally; in the UK numbers are projected to increase by 209,600 new cases per year [1]. The management of “behaviour that challenges” - the behavioural and psychological symptoms of dementia (BPSD) - which includes symptoms such as agitation, aggression, pacing, depression and hallucinations is a key challenge in dementia care pathways [2] [3]. There are significant costs associated with the management of BPSD [4]. BPSD is also called challenging behaviour or behaviour that challenges and is defined as being ‘any behaviour considered antisocial within the care environment or deemed dangerous to the person with dementia, their fellow residents, and staff’ [5].

Historically, antipsychotics have been used to treat BPSD [2]. The Banerjee Report found that antipsychotics were implicated in the death of 1800 people living with dementia; they also potentially worsen quality of life [3]. Antipsychotics were frequently used as a first-line treatment for behaviour that challenges that could be managed by other approaches and two thirds of their use may be inappropriate [3] [6]. Solely focussing on the prescribing of antipsychotics may simply drive prescribing to other, equally risky, psychotropics (such as anti-depressants and benzodiazepines), which also may worsen quality of life [7] [8] [9]. Research should test interventions to limit the use of all psychotropics [10] [11].

Prescribing within care homes in the UK is usually undertaken by General Practitioners (albeit possibly following a secondary care review), with medication supplied under the direction of a community pharmacist. Pharmacist-led medication review in residential homes may reduce prescribing of sedative and anticholinergic drugs [12]. In the UK the need for more pharmacy support in care homes has been identified [13]. Secondary care specialist dementia pharmacists may have a vital role in ensuring the appropriate treatment of BPSD [3] [14].

At least 300,000 people with dementia in the UK live in a care home; these people have complex needs [15] [16]. The need for training care home staff including helping with coping strategies and job stress reduction has been identified [17] [18]. A systematic review found that staff training could improve the well-being of staff working in dementia care, however the studies were variable in quality [18]. The Well-being and Health for People with Dementia (WHELD) study, which evaluated the impact of antipsychotic review and a wellbeing programme, concluded that a reduction in antipsychotic use can be achieved but non-pharmacological interventions are needed in parallel [19] [20].

This feasibility study involved a full clinical medication review by a specialist dementia care pharmacist combined with a health psychology informed behavioural change training intervention for care staff. The study aimed to provide key information on both study processes and outcomes, so that we could understand the challenges in evaluating the intervention [21]. This paper focusses on the feasibility of measuring clinical outcomes and intervention costs; recruitment and retention including implementation of the medication reviews are reported elsewhere [22].

### Aim

To determine whether it is feasible to measure the clinical outcome and costs of a dual purpose pharmacy–health psychology intervention incorporating medication review and staff training to limit the prescription of psychotropics to manage behaviour that challenges in care home residents.

## Methods

### Study design

An open label (non-blinded), mixed methods feasibility study, set within the MRC framework for developing a complex intervention was conducted [23]. The methods have been described elsewhere [24]; the full protocol is also available on www.aston.ac.uk/medrev. Briefly, the intervention contained two key elements: a medication review by a specialist dementia care pharmacist and training for care staff.

### Ethics

Ethical approval was received from the National Research Ethics Service (15/EM/0314).

### Setting

The study was conducted in five care homes in the West Midlands UK, each with at least 40 residents including people living with dementia from January 2015 until December 2017.

### Study participants

Care homes in the West Midlands were eligible. Residents in care homes were eligible if they had a diagnosis of dementia (e.g. on the GP dementia register) and were receiving medication for behaviour that challenges [including but not limited to medication in sections 4.1 (Hypnotics and Anxiolytics), 4.2 (Drugs used in psychoses and related disorders), 4.3 (anti-depressants) and 4.11 (Drugs used for Dementia) of the British National Formulary edition 68].

### Study procedures

#### Identification and recruitment of care homes

Care Homes meeting the inclusion criteria were identified from the Care Quality Commission (CQC) and other web-sites, and via the Enabling Research in Care Homes (ENRICH) initiative.

#### Recruitment of residents

People meeting the inclusion criteria, or their personal consultee, were approached regarding participation. The resident’s GP also had to consent to the medication review. Written consent/assent was obtained from the participant, or their personal consultee, as appropriate.

The target sample size was 45. This sample size was based on the number required to estimate the standard deviation for a subsequent sample size calculation [25] [26].

### Intervention

A full clinical medication review was conducted by a specialist dementia care pharmacist with the GP, the person with dementia and their carer as per detailed protocol [27]. The behavioural intervention consisted of a 3-h training session for care staff promoting person-centred care and brief training for primary care staff primarily on the treatment of BPSD [22].

### Outcome measures

Clinical Studies Officers (CSOs who undertake operational roles in research including data collection and obtaining consent) from the local research network conducted outcome assessments. The primary outcome measure was the Neuropsychiatric Inventory-Nursing Home version (NPI-NH) at 3 months after the medication was changed, or if the medication wasn’t changed the date of the initial baseline. This was completed by a Clinical Studies Officer questioning a member of care home staff, who knew the resident well [28] [29]. Other outcomes included quality of life (EQ-5D/DEMQoL) [30] [31], cognition (Standardised Mini Mental State Examination [sMMSE]) [32], service utilisation (modified version of Client Services Receipt Inventory [CSRI]) [33] and prescribed medication (including implementation of the review).

Data were collected at 8 weeks, and at 3 and 6 months after the medication was changed/date of initial baseline. Outcomes related to the feasibility included the feasibility of collecting clinical and cost-effectiveness data [e.g. response rates for each of the measures (reported here)], and recruitment and retention [22].

A qualitative evaluation exploring experiences and expectations of care home staff was conducted at the end of the study, and will be reported separately. The chief investigator (IM) also collected reflective comments from care staff and members of the research team to understand barriers and facilitators to participation and implementation of the intervention to inform any future trial, and if relevant these are reported below.

Care home staff who attended the training completed the Approaches to Dementia Questionnaire (pre-training, immediately and 3 months post-training) [34] and the Maslach Burnout Inventory – Human Services Survey (pre- and 3 months post-training) [35]; (this will be reported in a subsequent paper focussed on the Health Psychology components of the intervention including stakeholder accounts and results from the measures).

### Health economics

The health economics component sought to measure the levels of participant resource use and quality of life, to guide how costs and benefits could be measured in any future definitive study. Reported costs are based on the 2015/2016 financial year.

#### Intervention costs

Care homes were provided with a three-hour training session promoting person-centred care delivered by a band 7 psychologist [£52 per working hour [36]], this was provided twice in each care home. The average cost per hour for those care home staff who received the training was estimated to be £15.00 (including non-staff costs).

Primary care staff, including GPs [£111 per working hour [36]], practice nurses [£36 per working hour [36]] and a pharmacist [£42 per working hour [36]] received a briefer version of the training. Training delivery was costed for a clinical psychologist principal [£62 per working hour [36]] lasting 15 min when delivered by phone and 30 min in person. No additional costs were included for GP trainees when they joined a GP for face-to-face training, for consumables, travel or preparation time.

Levels of resource-use associated with the medication review [including preparation e.g. accessing patient notes, discussions (if applicable) with the patient, other family members, and other staff in primary care/care homes and time to make recommendations (including associated paperwork)] were estimated by the pharmacist who delivered the intervention. Aforementioned unit costs were then applied to all reported staff times, excluding travel, and then summed to estimate total intervention costs. Travel times were not costed due to an oversight related to the way the pharmacist was asked to report the travel time on the form for each patient - the form did not explicitly enable the pharmacist to report whether the travel time related to more than one participant. Thus, it was unclear how many patients the reported travel times should be apportioned across (this could just be the one patient or all patients in the same care home if the associated work was conducted on the same day).

#### Other (non-intervention) costs

Research staff were asked to extract details of participant use of other health care related services from care home records and when required by discussion with care home staff. Participants were no longer followed up for various reasons including death and if the medication review was not conducted [for full details see [22]].

#### Outcomes

Proxy completion of both the EQ-5D-5 L [37] and DEMQoL [38] was sought, where the caregiver, a member of care home staff, was requested to respond “as if” they were the person with dementia. In line with the NICE Position statement [39] responses to the EQ-5D-5 L were converted into utility scores [a scale where zero is equal to death and one is full health [40]] using a mapping approach based on the three-level version [41]. Responses to the DEMQoL Proxy version were similarly converted into utility scores [42]. Follow up was at baseline, and at 3 and 6 months post-implementation of the medication review, in the absence of any of the aforementioned exceptions. Also, at 8 and 16 weeks post-baseline, if the medication review recommendation had not been implemented then the baseline measures were repeated (referred to as re-baselining).

In order to assess feasibility, response rates, for each of the measures was estimated as a percentage of those for whom an attempt was made to collect data (i.e. after excluding those who were not followed up for reasons explained above). For non-intervention costs we only looked at the response at baseline as it was considered that the availability of care home records should not vary over time. Response rates for the EQ-5D-5 L and DEMQoL were estimated at baseline, 3 month and 6 month follow up points. Mean utility scores were also reported at each of these time points, based on those with complete data, though it is noted that these figures should be treated with caution due to small numbers.

## Results

### Characteristics of the study population

Thirty-four care home residents were recruited to the study, of whom 29 underwent a medication review (85.3%). Medication reviews were not carried out for five participants due to participant death (*n* = 2), primary care engagement issues (*n* = 2), and the pharmacist being unable to access primary care records (*n* = 1). The mean age of the 29 study participants who received a medication review was 83.6 years (SD 9.3; range 66 to 100). Eighteen participants were female (62.1%) and the remaining 11 participants were male (37.9%). The majority were White British in ethnicity (*n* = 24; 82.8%), with four participants from non-white ethnic groups (13.8%) (ethnicity data missing for one participant).

### Recommendations from medication reviews

A specialist dementia care pharmacist recommended stopping or reviewing a medication in 21 of the 29 participants who received a medication review (72.4%; see Table 1). Fifteen of the 21 recommendations involved antidepressants prescribing (71.4%; see Table 1).

**Table 1:** Details of medication involved in medication reviews (*n* = 21)

Details of Medication	Number
Citalopram	6
Sertraline	4
Mirtazapine	4
Anti-histamines	3
Trimipramine	1
Amisulpride	1
Co-prescribing of two hypnotics	1
Alendronic acid	1

### Feasibility of clinical evaluation

The primary outcome measure was the difference in NPI-NH total score between baseline (T0) and 3 month follow-up for participants whose medication had changed, compared to those whose medication did not change. A reduction of 4 points is considered a clinically important improvement/difference [28]. Table 2 summarises mean NPI-NH total scores for participants.

**Table 2:** Mean NPI total scores at each time point for participants whose medication changed vs. those where medication remained the same

	Baseline −2	Baseline −1	Baseline T0	8 week follow-up	3 month follow-up	6 month follow-up
*Participants with a medication change*						
Number of participants	*n* = 5	*n* = 11	*n* = 12	n = 11	*n* = 10	n = 5
Mean score (SD)	34.8 (30.5)	18.4 (19.7)	17.4 (13.9)	23.6 (22.6)	18.3 (12.3)	10.4 (8.4)
Range	10 to 70	0 to 71	0 to 51	0 to 77	0 to 33	0 to 18
*Participants with no medication change*						
Number of participants	*n* = 0	n = 0	*n* = 17	*n* = 13	*n* = 7	*n* = 4
Mean score (SD)	N/A	N/A	22.3 (24.0)	23.5 (28.7)	45.1 (32.0)	56.3 (39.7)
Range	N/A	N/A	0 to 83	0 to 59	22 to 120	0 to 114

Note: T0 is the baseline common to all participants, who received the medication review. Baseline −1 and − 2 refer to any previous baselining

For a number of participants there were several baseline measurements due to the need to re-baseline participants where recommendations had not been implemented by the 8-week follow-up.

Overall, it appeared feasible to measure the clinical effectiveness of the intervention using the NPI-NH; data were available for all participants who were still in the study at each time point. For the group whose medication changed, there was an increase of 0.9 points in NPI total score from baseline to 3 months (mean at T0 = 17.4, SD = 13.9; mean at 3 months = 18.3, SD = 12.3). For those whose medication did not change, there was an increase of 22.8 points (mean at T0 = 22.3, SD = 24.0; mean at 3 months = 45.1, SD = 32.0; *p* = 0.03).

Cognitive impairment was measured using the sMMSE. However, in practice, this measure did not prove feasible, as the majority of participants could not be rated using the measure at one or more time points during the study. Taking all time points together, data collection using the sMMSE was attempted on 88 occasions, yet study participants could be rated using the sMMSE in only 16 instances (18.2%). The Clinical Study Officers took steps to address potential reasons for non-completion. Some of these steps included having a quiet private space, the presence of a helpful relative and trying different times of day, particularly if “sun-downing” was evident. Clinical Study Officers reported that attempts to administer the questionnaire in participants who were unable to respond could be distressing for participants and themselves.

### Feasibility of health economic evaluation

#### Intervention costs

Trainer and care home staff costs for 142 staff were estimated to be £1560 and £6390, respectively. Costs for training primary care staff were £217 for the trainer and £958 for those receiving the training. Total training costs were thereby estimated to be £9125, or £268.38 per participant when equally apportioned across the 34 recruited participants. The pharmacist who delivered the intervention estimated the levels of resource-use associated with the medication review for all (100%) of the 29 participants who received a medication review. The mean pharmacist time per participant was 140 min; further costs included consulting care home staff for 23 of these participants (mean time = 7 min) and nurses for three participants (mean time = 3 min), and pharmacist follow-up for seven participants (mean time = 4 min). In total, the mean cost of the staff time associated with the medication review and the intervention (training plus medication review) was £104.41 and £372.80 respectively per participant.

However, there is potential for some of the above intervention costs to be off-set by savings elsewhere. For example, some GPs mentioned that the training intervention increased the confidence of care home staff to manage BPSD non-pharmacologically without the need for GP involvement. One GP stated that following the study, calls on the surgery had reduced by about one per week.

#### Other (non-intervention) costs

Levels of resource use for non-intervention costs (the CSRI) were available at baseline for the 29 participants who received a medication review; indicating 100% completion rates. For both the EQ-5D-5 L and DEMQoL, baseline data was obtained for 27 of the 29 participants, who received a medication review (93.1% response rate). At the 3 month follow up point 88.2% of those for whom it was requested responded (15/17), compared to 100% at the 6 month follow up point (9/9).

For both the EQ-5D-5 L and DEMQoL, 9 participants had complete data at each of the baseline, 3 month and 6 month follow up points. The mean scores for the EQ-5D-5 L, for these 9 participants, tended to be lower than for the DEMQoL [0.293 (range: − 0.095 to 1), 0.260 (− 0.358 to 1) and 0.280 (− 0.028 to 1) respectively, compared to 0.615 (0.538 to 0.672), 0.661 (0.412 to 0.900) and 0.662 (0.412 to 0.900) respectively]. Informal feedback from the Clinical Study Officers who collected the data was that the EQ-5D-5 L was more straightforward to rate.

## Discussion

A number of studies have investigated psychosocial interventions in care homes for behaviour that challenges [19] [43]. However, there is little research on the feasibility and effectiveness of medication review for care home residents [44] [45]. One trial has investigated a PCT/CCG-led medication review, but this did not have a specific focus on behaviour that challenges [46]. To the best of our knowledge, MEDREV is the first study to investigate the impact of a psychosocial intervention plus specialist medication review on behaviour that challenges.

Overall, the NPI-NH appeared to be a suitable tool to assess the clinical effectiveness of the intervention.

This confirms other studies; the WHELD intervention improved the total NPI-NH compared to Treatment as Usual by a mean difference of 4.55 (Standard Error of Mean [SEM] 1.28; 95% CI − 7.07,− 2.02; Cohen’s *D* 0.30) [19]; a similar result to MEDREV. Only a single measurement-instrument was used to assess neuropsychiatric symptoms in MEDREV, unlike some other studies, which used both the NPI and the Cohen-Mansfield Agitation Inventory [19] [47]. However, the Cohen-Mansfield may lack sensitivity to assess the broad range of neuropsychiatric symptoms [47].

The results build on consensus recommendations for core outcome measures for interventions to prevent or slow the progress of dementia for people living with mild to moderate dementia which recommend the NPI for measurement of neuropsychiatry symptoms [48]. Based on MEDREV and other studies, this recommendation could be extended to complex interventions to manage behavioural symptoms, and for people with moderate to severe dementia. If only a single measurement-instrument was used to rate such symptoms, this would reduce participant burden and costs.

The sMMSE lacked utility in this study; due to the degree of cognitive impairment it was only able to be rated in less than 20% of instances. This was despite taking steps to ameliorate the reasons for non-completion. The WHELD project used the Clinical Dementia Rating, an assessment of global deterioration as an outcome measure [19]; other studies have used both the sMMSE and the Severe Impairment Battery (SIB) [49]. The consensus guidance recommended the use of MMSE or the Alzheimer’s Disease Assessment Scale-Cognitive Scale [48]. However, the guidelines focus on assessing interventions such as cholinesterase inhibitors and disease modifying agents for mild to moderate dementia.

The research burden would be further simplified if a single tool was used to rate quality of life. Informal feedback from the Clinical Study Officers who collected the data favoured EQ-5D-5 L; this is also the tool recommended by NICE for health economic evaluations [50]. Other studies and the consensus guidance recommend DEMQOL [19] [48]. Both measures had good completion rates, however a noteable difference was that the DEMQOL scores tended to be higher, but cover a smaller range. This suggests that there is potential for different utility measures to give different results, as has been found previously [51] [52].

The cost of the intervention was substantial at approximately £100 per medication review, and training costs of £270 per participant; although this might be offset by savings including reduced GP workload. Furthermore, agitation – a key symptom of BPSD – is associated with significant costs; one-point increase in the NPI agitation score has been estimated to increase costs by £1064 over 12 months [4]. A recent Cochrane review found that the effect of interventions to optimise prescribing for older people in care homes on costs was mixed; three studies found a reduction in costs whereas two studies found no effect [53]. It cost £8627 per home involved to deliver the WHELD intervention [19].

In MEDREV, specialist dementia care pharmacist found that over 72% of residents with dementia recruited to the study were receiving unnecessary medication for BPSD (mainly anti-depressants). This may reflect the effectiveness of earlier national efforts to reduce antipsychotic prescribing for this target population, or the high level of performance of care homes interested in the research question. The evidence-base for anti-depressants is limited; the largest published study found an absence of benefit and an increased risk of adverse events, and concluded that the routine use of antidepressants in people with dementia should be reconsidered [54].

### Limitations

This was a feasibility study and only involved small numbers in a single area of the UK. The mean scores in particular in Table 2 should be treated with caution due to these small numbers and because the scores were based on different numbers at each time point. However, partly because of the ethics committee approved amendment on re-baselining, the outcome measures were rated on a significant number of occasions adding further weight to the feasibility findings regarding the use of these measures. The primary outcome for the study was the change in NPI-NH total score and the feasibility of collecting data on this. We have not included data on the NPI-NH sub-scales (agitation, apathy etc.) due to the small number of cases, but doing so would be a priority in any subsequent definitive trial of medication reviews that includes data collection using the NPI-NH instrument.

The protocol approved by the ethics committee stated that GP approval was required to enter residents into the study and for the medication review to be conducted. Participants (*n* = 3), for whom this approval was not obtained, were withdrawn from the study before baseline data was collected. Following an amendment, again approved by the ethics committee, if the GP did not implement the recommendations from the review (*n* = 6), residents were removed from the study and did not form part of the intention to treat analysis. As with any complex intervention, it was not clear the relative impact on the outcomes of the different components, in this case the medication review and staff training. Additionally, staff caring for participants in the no medication change group still received the training intervention.

The majority of participants were white and the findings on the feasibility of the outcome measures require confirmation in other populations. The sMMSE is an adaptation of the Mini Mental State Examination (MMSE), which has commonly been reported to have cultural and educational biases [55] [56]. However, even in this predominantly white population, the sMMSE was not able to be rated in the majority of instances.

The health resource use questionnaire asked about resource use in the previous 3 months. It was not clear how this was completed when the re-baseline time periods meant that this time period did not apply and there may be duplication or time gaps. This coupled with the follow up exceptions means that it is difficult to estimate a common cost time period. As such only completion at baseline is reported.

### Policy implications

Care home research is a key NIHR priority and future research should build on the current evidence base [45]. Furthermore, any interventions developed and the approach to developing interventions should be grounded in the reality of undertaking research in the care home setting [45]. Care homes are busy environments and staff time is precious, therefore research should aim to place the minimum burden on the home, staff and residents [57]. Based on the findings from this feasibility study, the assessment burden could and should be simplified, as much as is practical. Reflective comments also identified that researchers particularly Clinical Study Officers, who were collecting data, needed to be flexible and adaptive to the care home environment. This flexibility included collecting data at a time convenient for the home, avoiding peak times such as meal times and medication rounds.

### Future research

In similar future studies, the assessment burden should be rationalised; only a single quality of life measure, perhaps the EQ-5D-5 L, should be included. The sMMSE did not provide any useful data. Like other studies on behavioural symptoms, the NPI-NH appeared to be a suitable tool to assess the impact of a specialist medication review targeting all psychotropics. Future research on the appropriate treatment of BPSD should include the usage of all psychotropics and not solely focus on anti-psychotics.

## Conclusion

It was feasible to measure the clinical effectiveness of a complex intervention for behaviour that challenges in people with moderate to severe dementia using the NPI-NH, but not the sMMSE. It was also feasible to measure the impact of the intervention on quality of life, and the costs associated with the intervention and other non-intervention costs. The intervention shows some promise in ensuring the appropriate use of all psychotropics, but further research is required.

## Data Availability

The datasets used and/or analysed during the current study are available from the corresponding author on reasonable request.

## Abbreviations

BPSD: Behavioural and Psychological Symptoms of Dementia
CCG: Clinical Commissioning Group
CQC: Care Quality Commission
CSRI: Client Services Receipt Inventory
ENRICH: Enabling Research in Care Homes an NIHR research ready initiative for care homes in the UK
GP: General Practitioner
NPI-NH: Neuropsychiatric Inventory-Nursing Home version
PCT: Primary Care Trust
sMMSE: Standardised Mini Mental State Examination
